# An important role of interleukin-10 in counteracting excessive immune response in HT-29 cells exposed to *Clostridium butyricum*

**DOI:** 10.1186/1471-2180-12-100

**Published:** 2012-06-08

**Authors:** Quanxin Gao, Lili Qi, Tianxing Wu, Jinbo Wang

**Affiliations:** 1 Key Laboratory for Molecular Design and Nutrition Engineering, Ningbo Institute of Technology, Zhejiang University, No. 1 Qianhunan Road, Ningbo, 315100, China; 2 College of Animal Sciences, Zhejiang University, No. 388 Yuhangtang Road, Hangzhou, 310000, China

## Abstract

**Background:**

*Clostridium butyricum* has become increasingly important in preventing and treating intestinal inflammation. In the intestine it may increase the resistance of the gut to pathogen invasion via inducing the secretion of anti-inflammatory cytokines. Interleukin 10 (IL-10) plays a central role in preventing certain inflammatory diseases by down-regulating inflammatory cascades. In a previous study, we observed that the level of IL-10 mRNA was modulated by *C. butyricum*. The aim of this study was to investigate whether *C. butyricum* achieves its beneficial effects through IL-10.

**Results:**

We treated HT-29 cells with anti-IL-10 (IL-10 antibody) or siIL-10 (IL-10 small interfering RNA) to disrupt IL-10. In both cases, the effects of *C. butyricum*-induced NF-κB activation and IL-8 expression were enhanced. We also found that neutralization or knockdown of IL-10 could induce apoptosis and necrosis of HT-29 cells treated with *C. butyricum* compared with control cells.

**Conclusions:**

These findings show that IL-10 serves an important role in *C. butyricum-*mediated immune protection, and in host recognition of *C. butyricum*.

## Background

Probiotic bacteria are live microorganisms which are beneficial to the host organism, and can exert health benefits beyond those of inherent basic nutrition. A recent study indicates that the use of probiotics is rapidly advancing from the field of nutrition towards therapeutic applications [1]. Probiotics have proven useful in preventing and treating diarrhea. Crohn’s disease and ulcerative colitis patients exhibit loss of immune tolerance to enteric bacteria. Probiotics have modest but consistent prophylactic efficacy and can regulate innate and adaptive immunity to enhance innate defenses against microbes and maintainimmune homeostasis [2,3]. Therefore, immune modulation and inhibition of excessive immune response and inflammation are proposed to be mechanisms of action of probiotics [4,5].

It has been demonstrated that IL-10 plays a crucial role in down-regulating inflammatory cascades by suppressing the secretion of pro-inflammatory cytokines. IL-10-deficient mice develop chronic intestinal inflammation, which is in part caused by a loss of suppression of the mucosal immune response toward normal intestinal bacteria [6]. Recent studies have reported that topical treatment with IL-10 is effective in preventing certain inflammatory diseases. Moreover, probiotics can exert a therapeutic effect mediated through an IL-10-dependent mechanism [7]. It has been shown that oral administration of probiotics can prevent inflammation and mucosal ulcerations, which are associated with up-regulation of IL-10, which inhibits the increase of the CD4^+^ helper T cell population and down-regulates inflammatory cytokines [8].

Probiotics can exert immunomodulatory activities by increasing IL-10 production, which can in turn help prevent an excessive immune response. However, probiotic bacteria have multiple and diverse effects in the host, and not all probiotic strains act in this manner. The *C. butyricum* MIYAIRI II 588 stain has been used to prevent disturbances of microflora, treat diarrhea and enhance the humoral immune response in the human intestine [9]. However, the mechanisms by which *C. butyricum* treats and prevents diarrhea remain unclear. The aim of the present study was to assess whether *C. butyricum* achieves its beneficial effects via modulation of IL-10 production.

## Methods

### Bacterial strains and culture conditions

*C. butyricum* MIYAIRI II 588 used in this study was obtained from Miyarisan Pharmaceutical Co. Ltd, Tokyo, Japan. This strain is a butyric-acid producing, spore-forming and gram-positive rod bacterium [10]. It was cultured in MRS broth at 28°C in an anaerobic environment.

### Cell culture

HT-29 human colonic epithelial cells were purchased from the cell bank of the type culture collection of the Chinese academy of sciences (Shanghai). Enterocyte-like HT-29 cells were cultured in RPMI 1640 medium supplemented with 10% heat-inactivated fetal bovine serum, 100 U ml^−1^ penicillin, and 100 μg ml^−1^ streptomycin at 37°C in a humidified atmosphere of 5% CO_2_.

### SiRNA transient transfection

One day before transfection, HT-29 cells (1 × 10^6^ cells well^−1^) were allowed to attach and grow in 6-well culture plates (Corning, USA). When the plated cells in medium without antibiotics were 30–50% confluent, IL-10-specific siRNA (small interfering RNA) synthesized by Ribobio (Guangzhou, China) was transfected into cells with Lipofectamine 2000 (Invitrogen). After 48 h, cells were treated with *C. butyricum* and assayed for transfection efficiency by real-time PCR.

### IL-10 neutralization

IL-10 antibody-blocking was performed as described previously [11]. To prevent the effects of IL-10, supernatants were treated with IL-10 antibody (5 μg ml^−1^; HuaAn, China). These treated cells were then cultured in 6-well plates at 1 × 10^6^ cell well^−1^. After 48 h, the cells were stimulated with *C. butyricum*.

### Stimulation of cells

Before stimulation assays, the bacteria were collected and re-suspended in antibiotic-free 1640 media. To investigate whether *C. butyricum* regulates IL-10 expression in HT-29 cells, the cells were exposed to 1 × 10^6^, 1 × 10^7^, 1 × 10^8^ CFU ml^−1^ of *C. butyricum* for 2 h. The culture media were collected and analyzed for IL-10 by an enzyme-linked immunosorbent assay (ELISA), and the same cells from the original culture medium were harvested for real-time PCR analysis.

HT-29 cells pretreated with IL-10 antibody or siIL-10 were treated with 2 ml 1640 media or *C. butyricum* suspensions at designated concentration (1 × 10^8^ CFU ml^−1^), and incubated for 2 h. The culture media were collected and analyzed for IL-8 and IL-10 by ELISA, and the same cells from the original culture medium were harvested for real-time PCR and western blot analysis. In addition, we also detected the morphology of apoptotic cell nuclei using the PI method.

### Determination of IL-8 secretion using a sandwich ELISA

Human IL-8 proteins were assayed using BlueGene ELISA Kits, according to the manufacturer’s instructions (BlueGene Biotechnology, Shanghai, China).

### Western blot analysis for NF-κB (p50/105) and IκB expression

Total cellular and nuclear proteins were extracted according to the instructions of the nuclear and cytoplasmic protein extraction kit (Beyotime, Haimen, China). The nuclear extracts were used to determine NF-κB protein levels and the cytoplasmic extracts were used to determine IκB levels. The protein content of the lysates was estimated using an enhanced BCA protein assay kit (according to the manufacturer’s instructions). Fifty micrograms of protein from each sample were subjected to SDS-PAGE. After electrophoresis, proteins were electro-blotted to a Hybond-C Extra nitrocellulose membrane (Amersham, USA). The membrane was blocked at room temperature with 5% non-fat dry milk in TBS containing 0.3% Tween (TBS-T). The membrane was washed thrice with TBS-T and incubated overnight at 4°C with the primary antibody, anti-NF-κB (1:2000), anti-IκB (1:2500) and anti-*β*-actin (1:3000). This was followed by 1 h incubation with a 1:5000 dilution of the appropriate horseradish-peroxidase-conjugated secondary antibody. After incubation, the membrane was washed with TBS-T thrice. The antigen-antibody complexes were visualized by enhanced chemiluminescence and exposed to X-ray film for between 0.5 and 30 min [12].

### Real-time quantitative PCR

The cells were harvested and washed with ice-cold PBS. Total RNA was extracted using an RNA^TM^iso PLUS Kit (Takara Biotechnology, Dalian, China). The RNA was reverse transcribed into complementary DNA (cDNA) using PrimeScript 2st Strand cDNA Synthesis Kit (Takara Biotechnology, Dalian, China). Real-time cDNA amplification was performed using the SYBR Premix EX Taq^TM^ (Takara Biotechnology, Dalian, China). cDNA was then diluted 1:10 in RNase-free, diethyl pyrocarbonate-treated water. Table 1 shows the primers used for real-time quantitative RT-PCR. Amplification was carried out in 25.0 μl end volume containing 2 μl cDNA, 12.5 μl 2 × SYBR Premix EX Taq^TM^, 0.5 μl ROX Reference DyeII, 9 μl dH_2_O, and 10 μM of each primer. The amplification reactions were performed under the following PCR conditions: (i) one cycle at 95°C for 30 s, (ii) amplification including 40 cycles of 95°C for 10 s, 60°C for 20 s, (iii) 95°C for 30 s, 55°C for 1 min, 95°C for 30 s. The data represent mean values obtained in three independent experiments performed in duplicate.

**Table 1:** Oligonucleotide primers used to amplify RNA transcripts

**Primers**	**Forward primer (5′ to 3′)**	**Reverse primer (5′ to 3′)**
*β*-actin	CTA CAA TGA GCT GCG TGT GG	TAG CTC TTC TCC AGG GAG GA
IL-8	ATG ACT TCC AAG CTG GCC GTG GCT	TCT CAG CCC TCT TCA AAA ACT TCT C
IL-10	ATG CCC CAA GCT GAG AAC CAA GAC CCA	TCT CAA GGG GCT GGG TCA GCT ATC CCA

### Propidium Iodide (PI) assay

Morphology of apoptotic cell nuclei was detected by staining with the DNA binding fluorochrome PI (Beyotime Institute of Biotechnology, Jiangsu, China). The nuclei of apoptotic and necrosis cells were observed using fluorescence microscopy [13].

### Caspase-3 activity assay

The activity of caspase-3 was determined using the Caspase-3 activity Kit (Beyotime Institute of Biotechnology, Jiangsu, China). Cell lysates were prepared by incubating 2 × 10^6^ cells ml^−1^ in extraction buffer for 15 min on ice. After centrifugation at 20,000 × *g* for 15 min at 4°C, the supernatants were collected. In a 100 μl reaction volume, 10 μl sample or buffer (blank) were incubated with the substrate Ac-DEVD-*p*NA (acetyl-Asp-Glu-Val-Asp *p*-nitroanilide) in a 96-well microplate for 2 h at 37°C. The optical absorbance was measured at 405 nm using a microplate reader (A-5082, TECAN, Austria). Caspase-3 activity was expressed as the percentage of enzyme activity compared with the control [14].

### DNA fragmentation analysis

DNA was extracted using a DNA ladder extraction kit with spin column (Beyotime Institute of Biotechnology, Jiangsu, China). 10 μl of the DNA sample was separated on a 1.0% agarose gel and the DNA band pattern was visualized [14].

### Statistical analysis

All statistical analyses were performed using Statistical Analysis System software (SAS V8). All results are shown as the average of more than three replicates. Data are presented as mean ± the standard error (SE). Duncan’s multiple range tests were used to evaluate the statistical significance of the results. Differences with *p* values of < 0.05 were considered significant.

## Results

### *C. butyricum* stimulates elevated levels of IL-10 in HT-29 cells

**Figure 1:**
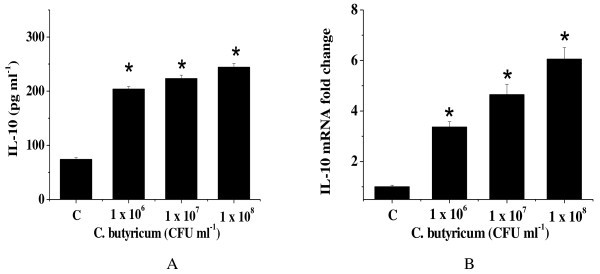
**IL-10 mRNA expression and IL-10 protein secretion were stimulated by*C. butyricum*.** The cells were exposed to 1 × 10^6^, 1 × 10^7^, 1 × 10^8^ CFU ml^−1^ of *C. butyricum* for 2 h. **(A)** At the end of the incubation period, cell culture supernatants were collected to determine IL-10 protein concentration by sandwich ELISA. **(B)** The same cells were harvested for real-time quantitative PCR. Data represent the mean ± the standard error of the mean for three experiments. *, *P* < 0.01 compared with the control. C: levels of IL-10 in control HT-29 cells.

To investigate whether *C. butyricum* regulates IL-10 expression in HT-29 cells, a stimulation assay was performed, as described in the methods. Figure 1A shows that IL-10 concentrations in the media of HT-29 cells cultured with *C. butyricum* were increased significantly. The same cells from the culture media were collected, and subjected to real-time PCR assay. In this case, IL-10 mRNA levels were also enhanced significantly by *C. butyricum* (Figure 1B). The correlation between the level of *C. butyricum* and IL-10 production or IL-10 mRNA expression was dose-dependent.

### Neutralization of IL-10 released by HT-29 cells enhances the effects of *C. butyricum*-induced NF-κB activation and IL-8 expression

**Figure 2:**
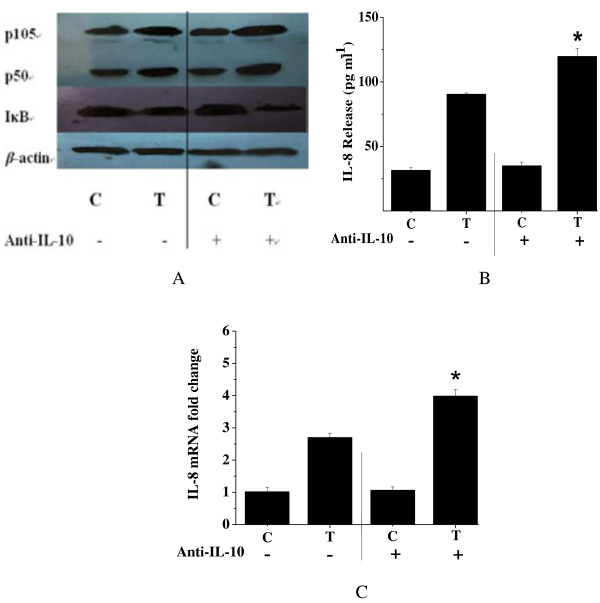
**Activation of NF-κB and up-regulation of IL-8 expression in HT-29 cells by*C. butyricum*were enhanced in the presence of IL-10 antibody.(A)** Immunoblot showing levels of NF-κB (p50/p105 subunits) and IκB in cells compared with the control. **(B)** IL-8 secretion in response to *C. butyricum* in control and anti-IL-10 treated cells. **(C)** IL-8 transcript levels as measured using real-time PCR. Results are mean ± SE for three experiments. *, *P* < 0.01 compared to the control without IL-10 antibody treatment (C- *vs*. C + and T- *vs*. T+). C: levels of NF-κB, IκB or IL-8 in control HT-29 cells. T: levels of NF-κB, IκB or IL-8 in HT-29 cells treated with *C. butyricum.*

Our previous study demonstrated that *C. butyricum* could induce HT-29 cells to release low levels of pro-inflammatory cytokines, which is similar to other probiotics such as *Lactobacilli*[15]*.* We also found that *C. butyricum* could increase the expression of anti-inflammatory cytokines, which may be associated with the beneficial properties of *C. butyricum.* In the current study, we have shown that *C. butyricum* can induce HT-29 cells to secrete IL-10. To determine whether this IL-10 present in culture supernatant affects the *C. butyricum*-induced immune response in HT-29 cells, an IL-10 antibody was utilized to treat HT-29 cells. Neutralization of IL-10 using anti-IL-10 for 48 h resulted in a significant degradation of cytoplasmic IκB protein and an increase in nuclear NF-κB and supernatant IL-8 levels (Figure 2). Therefore, it can be concluded that down-regulation of inflammatory cytokines and inhibition of excessive immunity in HT-29 cells induced by *C. butyricum* is probably mediated through IL-10.

### Knockdown of IL-10 enhances the effects of *C. butyricum*-induced NF-κB activation and IL-8 expression

**Figure 3:**
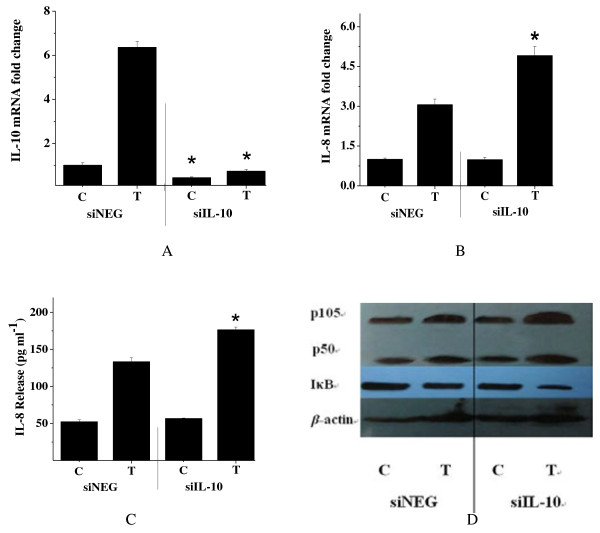
**SiRNA silencing of IL-10 enhances*C. butyricum*-induced NF-κB activation and IL-8 secretion.** HT-29 cells were transfected with siNEG (negative control-specific siRNA) or IL-10-specific siRNA for 48 h prior to stimulation. RNA was extracted after a 2 h *C. butyricum* treatment, and the levels of IL-10 **(A)** and IL-8 **(B)** were measured by real-time PCR. **(C)** IL-8 secretion in response to *C. butyricum* in siNEG control and IL-10 knockdown cells. **(D)** Immunoblot shows levels of NF-κB and IκB in cells with 20 nM IL-10 siRNA compared with the control. Results are mean ± SE for three experiments. C: levels of NF-κB, IκB or IL-8 in control HT-29 cells. T: levels of NF-κB, IκB or IL-8 in HT-29 cells treated with *C. butyricum*. *, *P* < 0.01 compared to the respective siNEG controls.

To further confirm the effects of IL-10 on the activation of NF-κB and secretion of IL-8, NF-κB, IκB and IL-8 levels were measured after pre-treating HT-29 cells with siNEG (negative control-specific siRNA) or siIL-10 (IL-10 small interfering RNA) for 48 h, and then treating them with *C. butyricum* for 2 h. The transfection of HT-29 cells with siRNA specific to IL-10 caused a significant reduction in IL-10 expression, confirming the efficient knockdown of the gene (Figure 3A). Similar to results obtained previously, IL-10 knockdown caused IκB degradation, NF-κB activation and increase in IL-8 expression (Figure 3B, C, D). These data confirmed our suggestion that *C. butyricum* achieves its beneficial effects on immune modulation through IL-10.

### Disruption of IL-10 induces apoptosis and necrosis of HT-29 cells with *C. butyricum*

**Figure 4:**
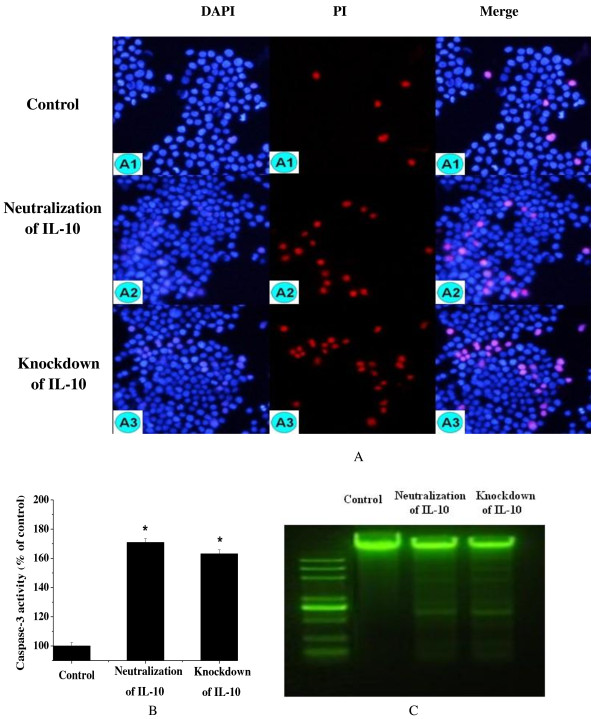
**Disruption of IL-10 induces apoptosis and necrosis of HT-29 cells treated with*C. butyricum*.** IL-10 antibody or siIL-10 treated cells were stimulated by *C. butyricum*. **(A)** After a 2 h incubation, cells were stained with DAPI and PI. Left: staining with DAPI; middle: PI immunocytochemistry; Right: merge of the two stains. A1, A2 and A3 indicated HT-29 cells of the control, IL-10 antibody and siIL-10 treated groups respectively. **(B)** Caspase-3 activation was measured using the chromogenic substrate Ac-DEVD-*P*na. **(C)** DNA fragmentation was detected using 1.0% agarose gel electrophoresis. M: 2,000 bp ladder markers. Control: the cells treated with *C. butyricum.*

The induction of apoptosis in intestinal epithelial cells by bacteria is well reported, and it may assist infection by pathogens [16]. The process of apoptosis is controlled by a diverse range of cell signals, which can be initiated by cytokines [17]. Following detection of enhancement of up-regulated NF-κB and IL-8 levels by disruption of IL-10, cell apoptosis and necrosis were observed after DAPI (4′,6-diamidino-2-phenylindole) and PI staining. DAPI is a fluorescent strain for labeling DNA that is commonly used to visualize nuclei and mitochondria. It can pass through an intact cell membrane, and can therefore be used on live or fixed cells. Apoptosis in late stage and necrosis can be detected using PI straining. A significant increase in the number of PI-positive cells (abnormal nuclei contents) in cells treated with IL-10 antibody or siIL-10 compared with the control was observed (Figure 4A). Furthermore, the activity of caspase-3 was also significantly increased (Figure 4B). In addition, DNA fragmentation was induced in the IL-10 antibody or siIL-10 treated cells (Figure 4C). These results indicate that lack of IL-10 can induce excessive immunity and even cell death in HT-29 cells.

## Discussions

The intestinal epithelial cell surface represents the largest exposed surface of the body that must be protected by the immune system against toxic substance and pathogenic bacteria. All intestinal epithelial cells are usually capable of regulating the immune response through different mechanisms, one of which is the secretion of anti-inflammatory cytokines. Throughout the present study, we have focused on the role of IL-10 in regulating epithelial cell function. IL-10 is a potent inhibitor of pro-inflammatory cytokine production, and has been shown to inhibit production of IL-6 and IL-1β in macrophages [18,19]. Supporting evidence for a role for IL-10 in inflammation is derived from studies in mice deficient in IL-10 or harboring mutated IL-10, which are a model of enterocolitis [20]. These IL-10^−/−^ mice under normal conditions show increased inflammatory responses and develop inflammatory bowel disease. Moreover, these IL-10^−/−^ mice are extremely susceptible to infection-induced immunopathology [21]. All these data suggest that endogenous IL-10 synthesis plays an important role in vivo in down-regulating immune responses and preventing host immunopathology. Moreover, beneficial effects in colitis patients have been obtained via probiotic bacteria-induced IL-10 production [22].

In our current study, *C. butyricum* stimulates elevated levels of IL-10 in HT-29 cells. Because this probiotic strain is frequently used in the management of allergic diseases or gastroenteritis, it is hypothesized that it promotes mucosal tolerance mediated through IL-10. Therefore, we further assessed the role of IL-10 in probiotic-mediated immune modulation by neutralizing or knocking down IL-10 in HT-29 cells. It was found that disruption of IL-10 enhanced effects of *C. butyricum*-induced NF-κB activation and IL-8 secretion. The results demonstrate that *C. butyricum* modifies the mucosal immune response to modulate the levels of specific molecules such as cytokines by increasing IL-10 levels and consequently decreasing inflammatory cytokines.

The viability of cells is dependent on cytokines. However, high-dose cytokines can induce apoptosis and necrosis. Bacteria and their metabolites can induce an anti-proliferative effect through induction of apoptosis [23-25]. In the current study, disruption of IL-10 enhanced *C. butyricum*-induced IL-8 secretion. We further assessed whether this probiotic strain induced apoptosis and necrosis of HT-29 cells due to a lack of effect of IL-10. The results showed that the number of abnormal cells significantly increased compared to the control, indicating that disruption of IL-10 caused a loss of suppression of the mucosal immune response and even excessive apoptosis and necrosis. This study confirmed that *C. butyricum* exerts anti-inflammatory effects and enhances tolerance to bacteria through increasing IL-10 production. Moreover, some subsets of Crohn’s disease patients are actually deficient in the production of IL-10, and therefore in this subset this probiotic therapy may be of limited use.

The adaptive co-evolution of humans and bacteria has resulted in the establishment of commensal relationships where neither partner is disadvantaged, or symbiotic relationships where both partners benefit [26]. In our current study, intestinal epithelial cells can secrete IL-10 to down-regulate inflammatory cascades through suppressing the secretion of pro-inflammatory cytokines. On the other hand, *C. butyricum* can drive the secretion of IL-10 to enhance tolerance to bacteria. Such mechanisms allow the host to recognize symbiotic bacteria without eliciting a deleterious immune response, and enable the symbiotic bacteria to reside in the gut, thus providing unique metabolic traits or other benefits. This pathway may be part of an evolutionarily primitive form of adaptive immunity.

## Conclusions

When HT-29 cells were pretreated with anti-IL-10 or siIL-10, *C. butyricum* induced an excessive immune response and even apoptosis and necrosis compared with control cells. These findings show that *C. butyricum* achieves its beneficial effects on immune modulation through IL-10. On the other hand, *C. butyricum* may have limited usefulness when the host is deficient in the production of IL-10; this requires further clarification.

## Authors’ contributions

QG and JW participated in the design of the experiment and its implementation, data analysis, and wrote the manuscript. LQ carried out bacteria culture, western blotting, real-time PCR and ELISA. TW was involved in the cell culture, SiRNA transient transfection, IL-10 neutralization, stimulation of cells, PI assay, Caspase-3 activity assay and DNA fragmentation analyses. All authors have read and approved the final manuscript. The authors declare no conflict of interest.
